# Annual Meeting of the New York State Medical Society

**Published:** 1870-02

**Authors:** 


### Annual Meeting of the New York State Medical Society.

The Society assembled in the Common Council Chamber, City Hall, Albany, February 1st, and after prayer was offered by Bishop Wm. C. Doane, the President, Dr. James P. White, of Buffalo, delivered the inaugural address, which is published entire in the foregoing pages of this Journal. Thanks were voted to Bishop Doane for his services, and to the President for his address, The following Committees were then announced:—On Arrangements, Drs. H. W. Dean, W. H. Bailey and N. C. Husted. On Business: Drs. Arnold, Whiton and Crandall. On Credentials: Drs. Bibbins, Odell and Townsend. On President's Address: Drs. Gray of Utica, Hutchinson of Brooklyn, and Porter of Albany. Dr. Bates of Lebanon Springs, reported as delegate to the State Medical Society of Vermont, Dr. Corliss to that of Illinois; Dr. Holbrook to New Hampshire; Dr. Crandall to New Jersey.

Dr. Squibb, on behalf of the Committee on Pharmacology, offered resolutions, which were adopted, appointing delegates to the Convention for Revision of the U. S. Pharmacopœia, viz:—Dr. Green, of Homer, Smith, of Manlius and Squibb, of New York, and agreeing to sustain a proportionate share of the expense of such convention. The titles of the following papers were announced by the Business Committee:—Compound Monstrosities, by Dr. Fisher of Sing Sing; Address before Chemung County Society, by Dr. Squire; On the relations of Fatty and Amyloid Degenerations, by Dr. S O. Vanderpoel; Cases of Chorea, by Dr. Wey; The Life and Character of the late Dr. March; An Address before the Albany Co., Society, by Dr. Babcock. The following papers were read: On a Case of Dislocated Tendons of the Peronei and Tibialis Posticus Muscles, caused by wearing high heeled boots; On Fracture of the Skull, by Dr. Curtiss; on certain Discolorations observed on the Cadaver, and a method of distinguishing them, by Dr. Porter; on the Management of Forceps, by Dr. Clarke; twelve cases of Trichiniasis, by Dr. Flandreau; on Nævi, by Dr. Hubbard; Chlorate of Potassa, by Dr. Peters; Colles' Fracture, by Prof. Moore of Rochester; Malignant Tumors of the Abdomen, by Dr. Hyde; Lithotomy and Lithotrity, by Dr. Gurdon Buck. Governor Hoffman sent an invitation to the members of the Society to a reception at the Executive Residence on Wednesday evening, which was accepted. The Governor and members of the Legislature belonging to the profession were invited to participate in the deliberations of the Society. The Committee on nominations, as announced by the President consisted of Drs. Quackenbush, Styles, Parker, Sherman, Dayton, Wey, Didamus and Wyckoff. The Committee on Ethics: Drs. Orton, Burdick and Doolittle. A telegram was received from the Minnesota State Medical Society, sending fraternal greeting and good wishes, which in a return telegram were cordially reciprocated. Dr. Allen, a delegate from the Pennsylvania State Society was introduced and kindly welcomed. Remarks were made by Dr. Arnold on a singular case of enlarged kidney, and also on the injuries given women by running sewing machines ; by Prof. Moore on Fracture of the Clavicle ; by Dr. Robert Newman, of New York, on the Endoscope; by Dr. Hutchinson, of Brooklyn, on a new instrument for rupturing the mucous membrane of the prepuce in phymosis, and by Dr. J. S. Hawley on Pepsin. Resolutions respecting the standard of Medical education were presented, discussed and laid upon the table. The honorary degree of doctor in medicine was voted to be conferred upon E. S. Lyman and Tobias J Greene. No prize was awarded the last year for essays, but it was moved that the Merrit H. Cash Prize be one hundred dollars for the coming year. A prize of one hundred dollars was offered by Dr. Hiram Corliss, the object of which will be announced hereafter. A resolution was passed requesting that each County Medical Society annually send to the editor of the Medical Register a list of the officers and other members of said County Society, together with their Post Office addresses, for publication in the Register. Dr. Garrish remarked upon the advantages of the hot douche to the os, for inducing premature labor in cases of deformed pelvis. Dr. Gray presented a remarkable case of hypospadia, and also one of cancer of the bladder, vagina and uterus. It was resolved that greater care should be taken in appointing permanent members; also that the society is of the opinion that instruction in cerebral and nervous diseases should be a part of the curriculum of the schools of medicine in the State; also that a committee consisting of Drs. Flint Elliot and Underhill be appointed to confer with that of the American Association respecting a nomenclature of diseases for the United States. A committee consisting of Drs. LaMores, Sayre and Boulware, to confer with the members of the Legislature respecting increasing the facilities for anatomical study was appointed. A communication from the Board of Regents of the University, announced their readiness to co-operate with the Society in efforts for the advancement of Medical education. The officers appointed for the ensuing year were then announced as follows :—Dr. S. O. Vanderpoel of Albany, President; Dr. Gibson A. Dayton, Mexico, Oswego Co., Vice President; Dr. Wm. H. Bailey of Albany, Secretary; Dr. John V. Lansing of Albany, Treasurer. The annual address by the President which was delivered on the evening of the second day of the meeting we hope to to be able to publish in next month's Journal.

